# An anti‐hair loss treatment in the management of mild androgenetic alopecia: Results from a large, international observational study

**DOI:** 10.1111/dth.15134

**Published:** 2021-10-22

**Authors:** Pascal Reygagne, Victor Desmond Mandel, Catherine Delva, Michaela Havlíčková, Kamila Padlewska, Rose Khalil, Veronique Meuleman, Gilberto Adame Miranda, Mariya Nevskaya, Jean‐Francois Michelet, Florence Pouradier, Sergio Vano‐Galvan, Delphine Kerob

**Affiliations:** ^1^ Centre Sabouraud Hôpital Saint‐Louis Paris France; ^2^ Dermatology Unit, Department of Clinical and Experimental Medicine University of Parma Parma Italy; ^3^ SyliaStat Lancrenon Bourg‐la‐Reine France; ^4^ Poliklinika Chmelnice Praha Czech Republic; ^5^ ORICEA Esthetic Dermatology & Anti‐Aging Medicine Warszawa Poland; ^6^ Dermatology Clinic Dahr Sarba Jounieh Sarba Lebanon; ^7^ Private Practice Tielt Belgium; ^8^ Del Valle Centro Ciudad de México, CDMX Mexico; ^9^ Trichology and Cosmetology Center of Tatyana Tsimbalenko Moscow Russian Federation; ^10^ L'Oréal Research and Innovation Aulnay‐sous‐Bois France; ^11^ L'Oréal Research and Innovation Saint‐Ouen France; ^12^ Trichology Unit. #TricoHRC Research Group, Department of Dermatology Ramón y Cajal University Hospital Madrid Spain; ^13^ Laboratoires Vichy International Levallois Perret France

**Keywords:** AC5, alopecia, androgenetic alopecia, female pattern hair loss

## Abstract

Androgenic alopecia (AGA) is a common and chronic condition. It may impact self‐esteem, self‐image and quality of life. Benefit, tolerability, cosmetic acceptance and patient satisfaction are key to ensure good treatment outcome. Hair loss improvement and hair quality with AC5 (2,4‐Diamino‐Pyrimidine‐N‐Oxyde, arginine, 6‐O glucose linoleate (SP94), piroctone olamine and Vichy mineralizing water) once daily was assessed in 527 subjects with mild AGA in an open‐label, observational, international real‐life study. After 3 months, investigators evaluated the impact of AC5 on hair loss, product satisfaction and asked subjects about local tolerance; subjects assessed hair growth and quality and satisfaction. Data from 357 subjects were evaluable for the benefit analysis; 59.9% of subjects were female; the mean age was 33.6±8.7 years. Duration of hair loss was 1.62±2.24 years. 71.3% of women had a Ludwig score of 1 and 40.8% of men had a Hamilton Norwood score of 2. At the end of study, hair loss was reduced in 89.0% of subjects; it was slightly higher in women (92.5%) than in men (83.8%). Subject satisfaction on a scale from 0 (not satisfied at all) to 10 (completely satisfied) was 7.9±1.7. Tolerance was rated good to very good by 98.6% of all subjects. In conclusion, AC5 reduces mild AGA in both men and women with a pleasant texture. AC5 was well tolerated and highly appreciated.

## 1 INTRODUCTION

Androgenetic alopecia (AGA) is a chronic scalp condition, representing the primary cause of hair loss in both women and men.^1^ The condition manifests in different clinical patterns among women and men. In women, AGA or female pattern of hair loss (FPHL) results in a decreased hair density and thickness, predominantly in the frontal area with a sparse pattern.^1^, ^2^, ^3^, ^4^, ^5^ Male pattern of hair loss (MPHL) presents predominantly in the temporal areas and the vertex more regularly than FPHL.^6^ In addition to the physical impact, alopecia also causes emotional distress, impacts the individual's quality of life, and can also be the first symptom of an underlying systemic disease.^2^, ^7^, ^8^, ^9^, ^10^ AC5 (Aminexil Clinical 5, Laboratoires Vichy, France), a marketed cosmetic product contains 2,4‐diamino‐pyrimidine‐N‐oxyde (DPNO), arginine, 6‐O glucose linoleate (SP94), piroctone olamine, and Vichy mineralizing water (VMW) as active ingredients. DPNO has shown a strong anti‐lysyl hydroxylase activity in vitro as well as its benefit in subjects with mild AGA.^11^, ^12^, ^13^, ^14^ Arginine was shown to improve microvascular function in the skin, piroctone olamine to increase hair shaft thickness, SP94 to fortify hair fibers, and VVMW to reduce skin inflammation.^15^, ^16^, ^17^, ^18^ Moreover, yet unpublished data have provided evidence that piroctone olamine, as VVMW, decreases inflammation.The aim of this cosmetic study was to assess hair loss improvement and hair quality with AC5 in subjects with mild AGA in real‐life settings.

## 2 METHODOLOGY

This cosmetic, open‐label, observational, international real‐life study was conducted according to the guidelines of the International Epidemiological Association for proper conduct in epidemiological research and in accordance with applicable regulatory requirements in Lebanon, Poland, Czech Republic, Russian Federation, Mexico, and Belgium, between October 2016 and February 2018. For participating European countries, subjects were informed of their rights with regard to the processing of their personal data through an information leaflet translated into their native language, in accordance with the Regulation (EU) 2016/679 of the European Parliament and of the Council of 27 April 2016 on the protection of natural persons. For countries outside the EU, local legal requirements for the conduct of this type of observational real‐world surveys were respected. Suitable subjects who were willing to participate were recruited by 75 dermatologists from six countries. The study planned for the inclusion of female and male subjects aged between 18 and 45 years with mild AGA. Mild AGA was defined for women as a score of 0 or 1 on the Ludwig scale and for men as a score between I and IV on the Hamilton‐Norwood scale. AC5 was applied alone or as an adjunct once daily on the scalp for 3 months using a specifically developed applicator device to stimulate the scalp and help the user to apply the product precisely. At the end of the study, the investigators evaluated the compliance of use and impact of AC5 on the subjects' hair loss on a four‐point scale (“worse,” “stable,” “slightly improved,” “clearly improved”), asked the subject to rate local tolerance of AC5 from “yes, very well” to “no, not at all,” and rated their product satisfaction on a scale ranging from “not at all satisfactory” to “highly satisfactory.” Subjects assessed their perception of hair growth and hair quality on an eight‐point questionnaire and their overall satisfaction on a visual analog scale (VAS) from 0 (not at all) to 10 (completely satisfied). Tolerability was assessed throughout the study. Statistical analyses were performed using SAS version 9.4. Qualitative variables were described as number and percent of the different response modalities; 95% confidence intervals were calculated, if necessary. Quantitative variables were described as number, mean, standard deviation, median, minimum, maximum, and number of missing data. Percentages were calculated based on the number of subjects who replied to each item. All statistical analyses were performed at a 5% significance level using two‐sided tests, except the normality tested at the threshold of 1% (Shapiro‐Wilk test).

## 3 RESULTS

**FIGURE 1:**
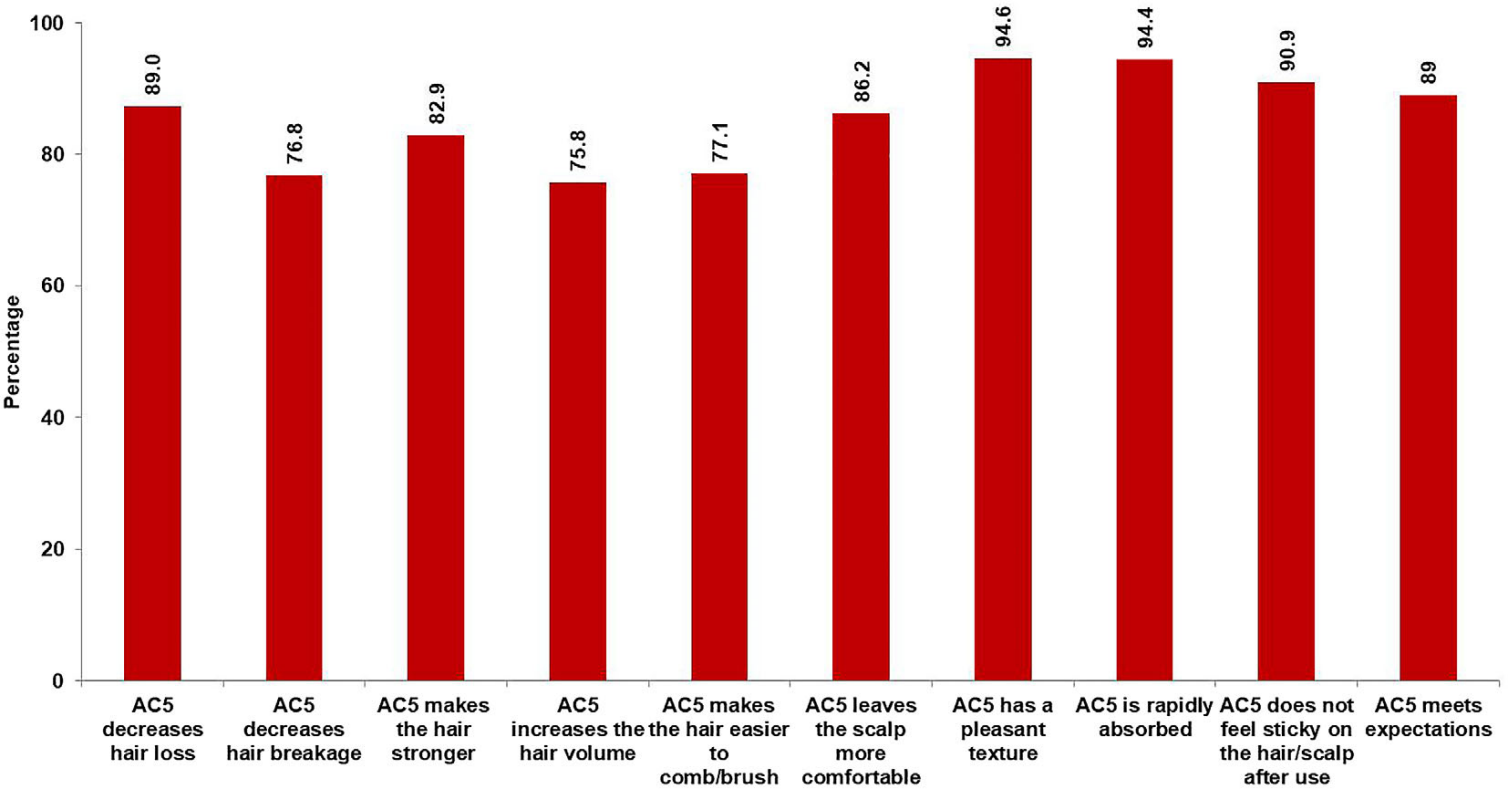
Subject perception after 3 months of daily application of AC5

Detailed demographic and baseline data are provided in Table 1. Overall, questionnaires from 527 subjects were collected. Only data from 357 subjects who did not use concomitantly other products to treat their AGA were evaluable for the statistical analysis of clinical efficacy. This was to assess the clinical efficacy of AC 5 alone. Data from the remaining subjects were not considered for analysis purposes. The main reasons for this were concomitant treatment of hair loss, insufficient treatment duration, and alopecia scores higher than I on the Ludwig scale or higher than four on the Hamilton‐Nordwood scale for men; moreover, for 14 subjects, questionnaires were incomplete. Other reasons were missing alopecia severity, inverted use of alopecia scales, wrong prescription; three subjects were aged less than 18 years. Overall, 59.9% (214/357) of subjects were women; the overall mean age was 33.6 ± 8.7 years. Duration of hair loss was 1.16 ± 1.70 years in women and 2.32 ± 2.73 years in men; overall mean duration was 1.62 ± 2.24 years. In total, 71.3% (176/247) of women had a Ludwig score of 1 and 40.8% (71/174) of men had a Hamilton Norwood score of 2. At inclusion, alopecia was progressive in 70.7% (251/355) of subjects more in men (83.3%, 115/138) than in women (62.7%, 136/217). Subjects applied AC5 for 82.9 ± 17.5 days; 94.8% (398/420) applied AC5 once daily while 5.2% (22/413) used it twice a day. Overall compliance was 95.9 ± 6.6%, with no notable difference between women and men. Dermatologists considered that hair loss had improved in 89.0% (315/354, CI95% [85.7; 92.2]) of subjects; it was somewhat better in women (92.5%, 196/212, CI95% [88.9; 96.0]) than in men (83.8%, 119/142, CI95% [77.7; 89.9]). The impact of AC5 on the subjects' hair quality was rated by the investigators as satisfactory or highly satisfactory for 92.4% (329/356, CI95% [89.7; 95.2]) of all subjects. It was slightly higher in women (94.4%, 202/214, CI95% [91.3; 97.5]) than in men (89.4%, 127/142, CI95% [84.4; 94.5]). Overall, 96.7% (59/61) of the investigators were satisfied with the benefit of AC5 in reducing the subjects' hair loss; all were satisfied with AC5 in daily clinical practice. A total of 98.4% (60/61) recommended AC5 for further use. AC5 met the subject's expectations in 89.0% (315/354, CI95% [85.7; 92.2]) with slightly more women (91.0%, 193/212, CI95% [87.2; 94.9]) than men (85.9%; 122/142; CI95% [80.2; 91.6]). Detailed results for subject rating are provided in Figure 1. The mean subject satisfaction score on the VAS scale was 7.9 ± 1.7; the difference to the average value (5 on the scale from 0 to 10) was significant (p<0.0001). Mean satisfaction was slightly higher in women (8.0±1.7, *p* < 0.0001) than in men (7.8±1.7, *p* < 0.0001). Tolerability was rated good to very good by 98.6% (502/509, CI95% [97.6; 99.6]) of all subjects.

**TABLE 1:** Demographic and baseline data (efficacy population)

Parameter	Female (*n* = 214)	Male (*n* = 143)	Total (*n* = 357)
**Gender (*n*, %); *n* = 357**	214 (59.9%)	143 (40.1%)	
**Age (years); *n* = 324** ^a^			
Mean ± *SD*	33.4 ± 8.8	33.9 ± 8.5	33.6 ± 8.7
Median	33.0	33.0	33.0
Min;max	18.0;68.0	18.0;59.0	18.0;68.0
**Duration of alopecia (years); *n* = 338** ^a^			
Mean ± *SD*	1.16 ± 1.70	2.32 ± 2.73	1.62 ± 2.24
Median	0.50	1.00	0.79
Min;max	0.1;12.00	0.1;12.00	0.1;12.00
**Age at onset (years); *n* = 309** ^a^			
Mean ± *SD*	32.1 ± 8.9	31.3 ± 8.6	31.8 ± 8.8
Median	31.4	30.8	31.0
Min;max	16.0;67.5	16.7;58.4	16.0;67.5
**Onset (*n*, %); *n* = 298** ^a^			
Acute	74 (39.2%)	18 (16.5%)	92 (30.9%)
Progressive	115 (60.8%)	91 (83.5%)	206 (69.1%)
**Alopecia care at inclusion; *n* = 357** ^a^			
Nonmedicated products	93 (43.5%)	39 (27.3%)	132 (37.0%)
*Topical products*	64 (29.9%)	29 (20.3%)	93 (26.1%)
*Oral products*	46 (21.5%)	13 (9.1%)	59 (16.5%)
**Previous treatments; *n* = 357** ^a^	42 (19.6%)	20 (14.0%)	62 (17.4%)
Alopecia Severity; *n* = 421	Ludwig scale L0: 71 (28.7%) L1: 176 (71.3%)	Hamilton‐Norwood scale N0: 6 (3.4%) N1: 19 (10.9%) N2: 71 (40.8%) N3A: 27 (15.5%) N3V: 33 (19.0%) N4: 18 (10.3%)	

^a^ Number of subjects for whom data were available.

## 4 DISCUSSION AND CONCLUSION

Results from this observational real‐life study show that AC5 combining DPNO Arginine and SP94 reduced hair loss in 89.0% of all subjects receiving AC5. Moreover, 89.0% of all subjects stated that they were satisfied or highly satisfied with their hair quality. Tolerability of AC5 was excellent, with similar results in the subset of the population not receiving any medical treatment at baseline. Moreover, as a result of a daily continual use over 3 months, investigators estimated that AC5 had a positive impact on the hair of more than 90% of all subjects. Subject satisfaction is a highly important element in the management of chronic diseases, as, for example, in other skin conditions, such as pruritus or psoriasis. In this real‐life observational study, satisfaction was high (almost 8 on the VAS of 10), and correlated well with reduced hair loss and improved hair quality, duly confirming that AC5 met the expectations of almost 90.0% of the subjects, even though alopecia is a difficult condition to manage.^19^, ^20^ In the past, several clinical studies assessed the benefit of DPNO alone, or in combination with other ingredients such as arginine or SP94. Loussouarn et al. assessed the clinical benefit of DPNO in six clinical studies involving a total of 351 male subjects compared with placebo.^13^ Four studies were conducted in spring and two in autumn, with an average duration of 3 and 6 months, respectively. Results from spring studies showed that the percentage of telogen hair in the DPNO group had significantly declined (*p* = 0.001), while this percentage had increased in the placebo group. Autumn studies showed that DPNO significantly improved hair density as early as after 6 weeks (*p* = 0.05) and led to improved growth after 6 months of daily use (*p* = 0.001). Piraccini et al. reported in an open label study with 118 subjects (52 men, 66 women), that when DPNO was combined with arginine and SP94, hair density in the vertex area increased by 7.8% and 5.5% and hair diameter thickness by 6.5% and 14.4% in men and women respectively, after 90 days of use. Hair loss efficacy was assessed by the pull test, with significant results in both men (−67.7%, [*p* < 0.01] at after 45 and −82% after 90 days [*p* < 0.01]) and women (−43.3% [*p* < 0.01] after 45 and −64.8% after 90 days [*p* < 0.01]). In men and women, the tested formulation reduced clinical signs (erythema and irritation) and symptoms (itching) by more than 70%.^11^More recently, Camacho et al. tested a combination of DPNO and SP94 in 180 female and male alopecia subjects.^12^ Results from this study provided evidence that subjects with alopecia and hyperseborrhoea benefit from the DPNO/SP94 combination with an increase of anagen hair of 10% and a decrease of telogen hair of 5%, however, with no change in sebum outflow and no improvement of signs and symptoms of seborrheic dermatitis. Yet unpublished in vivo data show that a formula containing aminexil 1.5% enriched with piroctone oleamide 0.25% and VVMW 1% reduces hair loss and significantly soothed skin in subjects with very sensitive skin, after 3 weeks of daily application. Results from these nine studies confirm that DPNO alone or combined with other ingredients such as arginine, SP94, piroctone olamine, or VVMW improves hair thickness and reduces hair loss and clinical signs and symptoms associated with AGA, even in subjects with more sensitive scalps. Thus, combining the individual benefit of each compound amplifies the clinical benefit of previous DPNO formulations and combinations. Today several other options to treat AGA exist, including minoxidil, 5‐alpha reductase inhibitors, their combinations, hormonal and more invasive therapies such as platelet‐rich plasma injections, and surgery.^21^ Minoxidil and 5‐alpha reductase inhibitors have been considered by the expert group to be the most efficacious to prevent mild AGA progression while 5‐alpha reductase inhibitors were considered the easiest to be used by subjects. Evaluations were based on published clinical data. Although several studies have been performed to prove the clinical efficacy of AC5 in alopecia, none of them had been yet published in peer‐reveiwed journals.^11^, ^12^, ^13^ However, we acknowledge that this study has several limitations. The main limitation is its observational study design which does not provide an objective comparison with a placebo or a comparative cosmetic product. Moreover, the study did not use objective clinical assessments as usually performed during clinical trials. As this was a large observational study conducted mostly in private practices and under real‐world conditions, assessments chosen were those commonly used by the investigators in their daily clinical practice. Using specific devices such as a phototrichogram to assess instrumentally the benefit of AC5 would, of course, have been preferential, but impossible to be put in place for each investigator. Nevertheless, we believe, that when regarding treatment outcomes observed in both women and men, AC5 used in monotherapy and under the study conditions confirmed that combining several ingredients, known for their clinical benefit, notably reduces hair loss.^15^, ^16^, ^17^, ^18^ Despite these limitations, AC5 improves hair loss and hair quality in subjects with mild AGA with an excellent tolerability, a pleasant texture, and a high satisfaction rate.

## CONFLICT OF INTEREST

Pascal Reygagne, Victor Desmond Mandel, and Sergio Vano‐Galvan are consultants of Laboratories Vichy International. Florence Pouradier, Jean‐Francois Michelet are employees of l'Oréal France. Delphine Kerob is an employee of Laboratoires Vichy International France. The other authors have no conflict of interest to disclose.

## AUTHOR CONTRIBUTIONS

Pascal Reygagne, Victor Desmond Mandel, Catherine Delva, Michaela Havlíčková, Kamila Padlewska, Rose Khalil, Veronique Meuleman, Gilberto Adame Miranda, Mariya Nevskaya, Sergio Vano‐Galvan, were experts for this study and recruited subjects. Florence Pouradier, Delphine Kerob, and Jean‐Francois Michelet wrote the protocole and conducted the study. All authors participated in writing the manuscript and all approved its content.

## Data Availability

The data that support the findings of this study are available from the corresponding author upon reasonable request.
